# New Insights Into the Regulatory Roles of Extracellular Vesicles in Tumor Angiogenesis and Their Clinical Implications

**DOI:** 10.3389/fcell.2021.791882

**Published:** 2021-12-13

**Authors:** Maohua Huang, Yuhe Lei, Yinqin Zhong, Chiwing Chung, Mei Wang, Min Hu, Lijuan Deng

**Affiliations:** ^1^ Formula Pattern Research Center, School of Traditional Chinese Medicine, Jinan University, Guangzhou, China; ^2^ College of Pharmacy, Jinan University, Guangzhou, China; ^3^ Shenzhen Hospital of Guangzhou University of Chinese Medicine, Shenzhen, China; ^4^ Department of Hepatobiliary Surgery, Jinan University First Affiliated Hospital, Guangzhou, China

**Keywords:** extracellular vesicles, tumor angiogenesis, miRNAs, lncRNAs, CircRNAs, proteins

## Abstract

Angiogenesis is required for tumor growth and development. Extracellular vesicles (EVs) are important signaling entities that mediate communication between diverse types of cells and regulate various cell biological processes, including angiogenesis. Recently, emerging evidence has suggested that tumor-derived EVs play essential roles in tumor progression by regulating angiogenesis. Thousands of molecules are carried by EVs, and the two major types of biomolecules, noncoding RNAs (ncRNAs) and proteins, are transported between cells and regulate physiological and pathological functions in recipient cells. Understanding the regulation of EVs and their cargoes in tumor angiogenesis has become increasingly important. In this review, we summarize the effects of tumor-derived EVs and their cargoes, especially ncRNAs and proteins, on tumor angiogenesis and their mechanisms, and we highlight the clinical implications of EVs in bodily fluids as biomarkers and as diagnostic, prognostic, and therapeutic targets in cancer patients.

## 1 Introduction

Angiogenesis, defined as the establishment of new blood vessels from pre-existing vascular networks, is triggered by proangiogenic factors and depends on the proliferation and migration of endothelial cells (ECs) (Teleanu et al., 2019; Lugano et al., 2020). In normal healthy tissues, angiogenesis is tightly regulated by a balance that is maintained between proangiogenic and antiangiogenic factors. Solid tumors are generally characterized with aberrant angiogenesis, and tumor angiogenesis is critically required for tumor growth and development (Teleanu et al., 2019; Lugano et al., 2020). Many proangiogenic factors are upregulated in tumor cells and tumor-associated stromal cells, including vascular endothelial growth factor (VEGF), fibroblast growth factor (FGF), and delta ligand-like 4 (Dll4). Hypoxia is a key inducer of tumor angiogenesis and promotes the expression of various proangiogenic factors in the tumor microenvironment (Abou Khouzam et al., 2020). Recently, antiangiogenic drugs have been widely applied to the treatment of multiple solid cancers, and cancer patients have gained tremendous survival benefits from antiangiogenic therapy.

Extracellular vesicles (EVs), such as microvesicles and exosomes, are nanosized vesicles with lipid membranes that are secreted by most cells. EVs contain many bioactive molecules, such as microRNAs (miRNAs), long noncoding RNAs (lncRNAs), circular RNAs (circRNAs), and proteins, and these EV cargoes regulate intercellular communication (Mathieu et al., 2019; Liu et al., 2021). Donor cell-derived EVs are taken up by recipient cells, and the encapsulated bioactive components are thus delivered to recipient cells, enabling their regulation of recipient cell biological behaviors. An increasing number of studies have demonstrated that EVs play important roles in tumorigenesis, tumor growth, metastasis, immune evasion, drug resistance, and angiogenesis (Todorova et al., 2017; Aslan et al., 2019). Tumor-derived EVs can transfer proangiogenic molecules into ECs to promote their angiogenic activity via various mechanisms such as VEGF/VEGF Receptor (VEGF/VEGFR), Notch, Wingless-type (WNT), and Hypoxia-inducible factor (HIF) signaling pathway (Phng et al., 2009; Horie et al., 2017; Todorova et al., 2017; Aslan et al., 2019). Thus, targeting EVs might be an innovative and promising therapeutic strategy to inhibit tumor angiogenesis.

A wide variety of biomolecules, including ncRNAs and proteins, have been identified as EV cargoes, and these signaling molecules can be transported from donor cells to recipient cells. To date, considerable attention has been directed to the effects of EVs on tumor angiogenesis and the clinical relevance of these effects. A database of exosomes (http://www.exocarta.org/) includes 9,769 proteins, 3,408 mRNAs, and 2,838 miRNAs. The mechanisms triggered by these specific cargos loaded into EVs and delivered from donor cells to acceptor cells are complex (Abels and Breakefield, 2016; Mathieu et al., 2019). This article summarizes the current knowledge on the roles of tumor-derived EVs in angiogenesis, with a particular emphasis on the molecular mechanisms involved. We also discuss the main prospects for their applications in cancer diagnosis, prognosis, and treatment.

## 2. Extracellular Vesicles and Tumor Angiogenesis

### 2.1 EV-Derived ncRNAs and Tumor Angiogenesis

Here, we focus on the effects and mechanisms of EV-derived miRNAs, lncRNAs, and circRNAs on angiogenesis, aiming to elucidate their potential as tumor biomarkers and therapeutic targets for tumor angiogenesis.

#### 2.1.1 miRNAs

Various miRNAs are packaged into tumor-derived EVs and can be transferred into recipient ECs (Muralidharan-Chari et al., 2009). Once internalized by ECs, these miRNAs can initiate an angiogenic switch by modulating EC proliferation and migration and regulating the expression of angiogenesis-related genes (Huang et al., 2020a; Li et al., 2020; Masoumi-Dehghi et al., 2020).

VEGF/VEGFR and HIF signaling pathways are the main targets of miRNAs that regulate angiogenesis. Exosomal miR-130a secreted by gastric cancer (GC) cells targeted c-MYB in ECs and promoted angiogenesis *in vitro* and *in vivo* (Yang et al., 2018). Similarly, GC cell-derived exosomal miR-155 downregulated c-MYB but increased the expression of VEGF in ECs, which enhanced EC tube formation and increased microvessel density in xenografted tumors (Deng et al., 2020). Moreover, inhibition of signal transducer and activator of transcription 3 (STAT3) reduced miR-21 levels in exosomes derived from transformed human bronchial epithelial cells, and these exosomes suppressed angiogenesis by blocking the STAT3/VEGF axis in ECs (Liu et al., 2016). MiR-182-5p in glioblastoma-derived EVs directly targeted Kruppel like factor 2 (KLF2) and KLF4, which resulted in VEGFR accumulation in ECs and thus promoted angiogenesis (Li et al., 2020). In addition, HIF is a critical angiogenesis inducer that regulates the cellular response to hypoxia-induced stress (Shao et al., 2018). Under hypoxic conditions, HIF-1α is stabilized and its expression is increased, which facilitates the expression of various proangiogenic factors (Horie et al., 2017). Tumor cell-derived exosomal miRNAs, such as miR-21-5p, miR-23a, miR-155, miR-181a, miR-182-5p, and miR-619-5p, were also upregulated under hypoxia. Prolyl hydroxylase (PHD) is a negative regulator of HIFs, and inhibition of PHD can induce the accumulation of HIFs in cells. Exosomal miR-23a derived from hypoxic lung cancer cells inhibited the expression of PHD1 and PHD2 and led to the accumulation of HIF-1α in ECs, thereby enhancing angiogenesis (Hsu et al., 2017).

EVs derived from tumor stromal cells, such as cancer-associated fibroblasts (CAFs) and tumor-associated macrophages (TAMs), can trigger tumor angiogenesis via various mechanisms. Tumor-derived EVs can promote the transformation of fibroblasts into CAFs and induce M2 polarization of macrophages, thereby inducing the proangiogenic macrophage phenotype switch. For example, lung cancer cell-secreted exosomal miR-210 activated the janus kinase 2 (JAK2)/STAT3 pathway by targeting ten-eleven translocation 2 (TET2) in fibroblasts and thus initiated the acquisition of the proangiogenic phenotype in CAFs, as indicated by the upregulation of VEGFA, MMP9, and FGF2 (Fan et al., 2020). TAMs are immune cells that play a significant role in tumor angiogenesis (Zheng et al., 2018) and M2 macrophages express high levels of proangiogenic factors such as VEGF (Corliss et al., 2016). M2 macrophages were associated with increased microvessel density in pancreatic ductal adenocarcinoma (PDAC) tissues, and exosomal miR-155-5p and miR-211-5p derived from M2 macrophages targeted E2F transcription factor 2 (E2F2) and promoted the angiogenic functions of mouse aortic ECs *in vitro* (Yang et al., 2021).

Collectively, miRNA-derived from tumor-secreted EVs regulate angiogenesis primarily by modulating the VEGF/VEGFR and HIF-1α signaling pathways. In addition to those derived from tumor cells, EVs derived from CAFs and TAMs have been shown to regulate tumor angiogenesis via various mechanisms. The effects and mechanisms of other EV-derived miRNAs on tumor angiogenesis are summarized in Table 1.

**TABLE 1:** The effects and mechanisms of miRNAs, lncRNAs, and circRNAs derived from tumor EVs on angiogenesis.

Cargoes	Cancer types	Recipient cells	Target genes or signaling pathways	Functions	References
MiRNAs					
miR-9	NPC	HUVECs	MDK, PDK/Akt pathway	Inhibition	Lu et al. (2018)
	Glioma	HUVECs	COL18A1, THBS2, PTCH1, PHD3, HIF-1α, VEGF	Promotion	Chen et al. (2019)
miR-17-5p	NPC	HUVECs	BAMBI	Promotion	Duan et al. (2019)
miR-21	ESCC	HUVECs	SPRY1	Promotion	Zhuang et al. (2020)
miR-21-5p	Hypoxic PTC	HUVECs	TGFBI, COL4A1	Promotion	Wu et al. (2019a)
miR-23a	Hypoxic HCC	HUVECs	SIRT1	Promotion	Sruthi et al. (2018)
	NPC	HUVECs	TSGA10	Promotion	Bao et al. (2018)
	GC	HUVECs	PTEN	Promotion	Du et al. (2020)
miR-25-3p	CRC	HUVECs	KLF2, KLF4, VEGFR2, ZO-1, Occludin, Claudin5	Promotion	Zeng et al. (2018)
miR-26a	Glioma stem cells	HBMECs	PTEN, PI3K/Akt pathway	Promotion	Wang et al. (2019c)
miR-27a	PC	HMVECs	BTG2	Promotion	Shang et al. (2020b)
	ccRCC	HUVECs	SFRP1	Promotion	Hou et al. (2021)
miR-92a-3p	Retinoblastoma	HUVECs	KLF2	Promotion	Chen et al. (2021a)
miR-130a	GC	HUVECs	c-MYB	Promotion	Yang et al. (2018)
miR-130b-3p	OSCC	HUVECs	PTEN	Promotion	Yan et al. (2021)
miR-135b	GC	HUVECs	FOXO1	Promotion	Bai et al. (2019)
miR-135b-5p	CAFs from CRC	HUVECs	TXINP	Promotion	Yin et al. (2021)
miR-141	SCLC	HUVECs	KLF12	Promotion	Mao et al. (2020)
miR-141-3p	EOC	HUVECs	SOCS5, VEGFR2, JAK/STAT3 and NF-κB signaling pathways	Promotion	Masoumi-Dehghi et al. (2020)
miR-148a-3p	Glioma	HUVECs	ERRFI1, EGFR/MAPK signaling pathway	Promotion	Wang et al. (2020b)
miR-155	GC	HUVECs	c-MYB/VEGFA axis	Promotion	Deng et al. (2020)
miR-155	GC	HUVECs	FOXO3a	Promotion	Zhou et al. (2019)
	Melanoma	fibroblasts	SOCS1/JAK2/STAT axis, VEGFA, FGF2, MMP9	Promotion	Zhou et al. (2018)
	Hypoxic HCC	HUVECs	—	Promotion	Matsuura et al. (2019)
miR-155-5p	M2 macrophages	MAECs	Targets E2F2 in PDAC	Promotion	Yang et al. (2021)
miR-221-5p					
miR-181a	Hypoxic PTC	HUVECs	DACT2, MLL3, YAP/VEGF axis	Promotion	Wang et al. (2021b)
miR-182-5p	Hypoxic GBM	HUVECs	KLF2, KLF4, VEGFR, ZO-1, occludin, claudin-5	Promotion	Li et al. (2020)
miR-183-5p	CRC	HMEC-1	FOXO1	Promotion	Shang et al. (2020a)
miR-205	OC	HUVECs	PTEN/Akt pathway	Promotion	He et al. (2019)
miR-210	LC	CAFs	JAK2/STAT3	Promotion	Fan et al. (2020)
	HCC	HUVECs	SMAD4, STAT6	Promotion	Lin et al. (2018)
miR-210-3p	OSCC	HUVECs	EFNA3, PI3K/Akt pathway	Promotion	Wang et al. (2020a)
miR-221-3p	CSCC	HUVECs	THBS2	Promotion	Wu et al. (2019b)
	CC	MVECs	MAPK10	Promotion	Zhang et al. (2019)
miR-378b	HCC	HUVECs	TGFBR3	Promotion	Chen et al. (2021b)
miR-549a	TKI-resistant ccRCC	HUVECs	HIF-1α, VEGF	Promotion	Xuan et al. (2021)
miR-619-5p	Hypoxic NSCLC	HUVECs	RCAN1.4	Promotion	Kim et al. (2020)
miR-944	Glioma stem cells	HUVECs	VEGFC, Akt, Erk1/2 signaling pathway	Inhibition	Jiang et al. (2021)
miR-1229	CRC	HUVECs	HIPK2, VEGF pathway	Promotion	Hu et al. (2019)
miR-1260b	NSCLC	HUVECs	HIPK2	Promotion	Kim et al. (2021)
miR-1290	HCC	HUVECs	SMEK1	Promotion	Wang et al. (2021a)
miR-3157-3p	NSCLC	HUVECs	TIMP2, KLF2, VEGF, MMP2, MMP9, occludin	Promotion	Ma et al. (2021)
miR-3682-3p	HCC	HUVECs	ANGPT1, RAS-MEK1/2-ERK1/2 signaling pathway	Inhibition	Dong et al. (2021)
LncRNAs					
LncRNA H19	Glioma	HBMVECs	miR-29a, VASH2	Promotion	Jia et al. (2016)
LncRNA H19	CD90^+^ liver cancer	HUVECs	VEGF, VEGFR, ICAM1	Promotion	Conigliaro et al. (2015)
LncRNA HOTAIR	Glioma	HBMVECs	VEGFA	Promotion	Ma et al. (2017)
LncRNA CCAT2	Glioma	HUVECs	VEGFA, TGFβ	Promotion	Lang et al. (2017b)
LncRNA POU3F3	Glioma	HBMECs	bFGF, FGFR, VEGFA, and ANG	Inhibition	Lang et al. (2017a)
LncRNA MALAT1	EOC	HUVECs	VEGFA, VEGFD, ENA78, PIGF, IL8, ANG, bFGF, Leptin	Promotion	Qiu et al. (2018)
LncRNA GAS5	LC	HUVECs	miR-29-3p, PTEN	Inhibition	Cheng et al. (2019)
LncRNA p21	NSCLC	HUVECs	miR-23a, miR-146b, miR-330, miR-494	Promotion	Castellano et al. (2020)
LncRNA UCA1	PC	HUVECs	miR-96-5p/AMOTL2/ERK1/2 axis	Promotion	Guo et al. (2020)
LncRNA RAMP2-AS1	Chondrosarcoma	HUVECs	miR-2355-5p/VEGFR axis	Promotion	Cheng et al. (2020)
LncRNA APC1	CRC	HUVECs	Rab5b, MAPK	Promotion	Wang et al. (2019a)
LncRNA TUG1	CC	HUVECs	—	Promotion	Lei and Mou, (2020)
LncRNA X26 nt	GC	HUVECs	VE-cadherin	Promotion	Chen et al. (2021c)
LncRNA OIP5-AS1	Osteosarcoma	HUVECs	miR-153, ATG5	Promotion	Li et al. (2021c)
LncRNA AC073352.1	BC	HUVECs	YBX1 stabilization	Promotion	Kong et al. (2021)
LncRNA SNHG16	HCC	HUVECs	miR-4500/GALNT1 axis, PI3K/Akt/mTOR pathway	Promotion	Li et al. (2021b)
LncRNA CCAT1	PC	HUVECs	miR-1138-5p/HMGA1 axis	Promotion	Han et al. (2021)
LncRNA LINC00161	HCC	HUVECs	miR-590-3p/ROCK axis	Promotion	You et al. (2021)
LncRNA SNHG11	PC	HUVECs	miR-324-3p/VEGFA axis	Promotion	Fang et al. (2021)
CicrRNAs					
Circ-100338	HCC	HUVECs	MMP9	Promotion	Huang et al. (2020b)
Circ-SHKBP1	GC	—	miR-582-3p/HUR/VEGF axis	Promotion	Xie et al. (2020b)
Circ-RanGAP1	GC	HUVECs	miR-877-3p/VEGFA axis	Promotion	Lu et al. (2020)
Circ-CCAC1	CCA	HUVECs	SH3GL2, EZH2, ZO-1, Occludin	Promotion	Xu et al. (2021)
Circ-0044366	GC	HUVECs	miR-29a/VEGF axis	Promotion	Li et al. (2021a)
Circ-CMTM3	HCC	HUVECs	miR-3619-5p/SOX9	Promotion	Hu et al. (2021)

Abbreviation: Breast cancer, BC; Cervical cancer, CC, Cervical squamous cell carcinoma; CSCC; Clear cell renal cell carcinoma, ccRCC; Cholangiocarcinoma, CCA; Colorectal cancer, CRC; Epithelial ovarian cancer, EOC; Esophageal squamous cell carcinoma, ESCC; Gastric cancer, Glioblastoma, GBM; GC; Hepatocellular carcinoma, HCC; Lung cancer, LC; Mouse aortic endothelial cells, MAECs; Nasopharyngeal carcinoma, NPC; Non-small cell lung cancer, NSCLC; Ovarian cancer, OC; Oral squamous cell carcinoma, OSCC; Pancreatic cancer, PC; Pancreatic ductal adenocarcinoma, PDAC; Papillary thyroid cancer, PTC; Small cell lung cancer, SCLC., Tyrosine kinase inhibitor, TKI.

#### 2.1.2 LncRNAs

**FIGURE 1:**
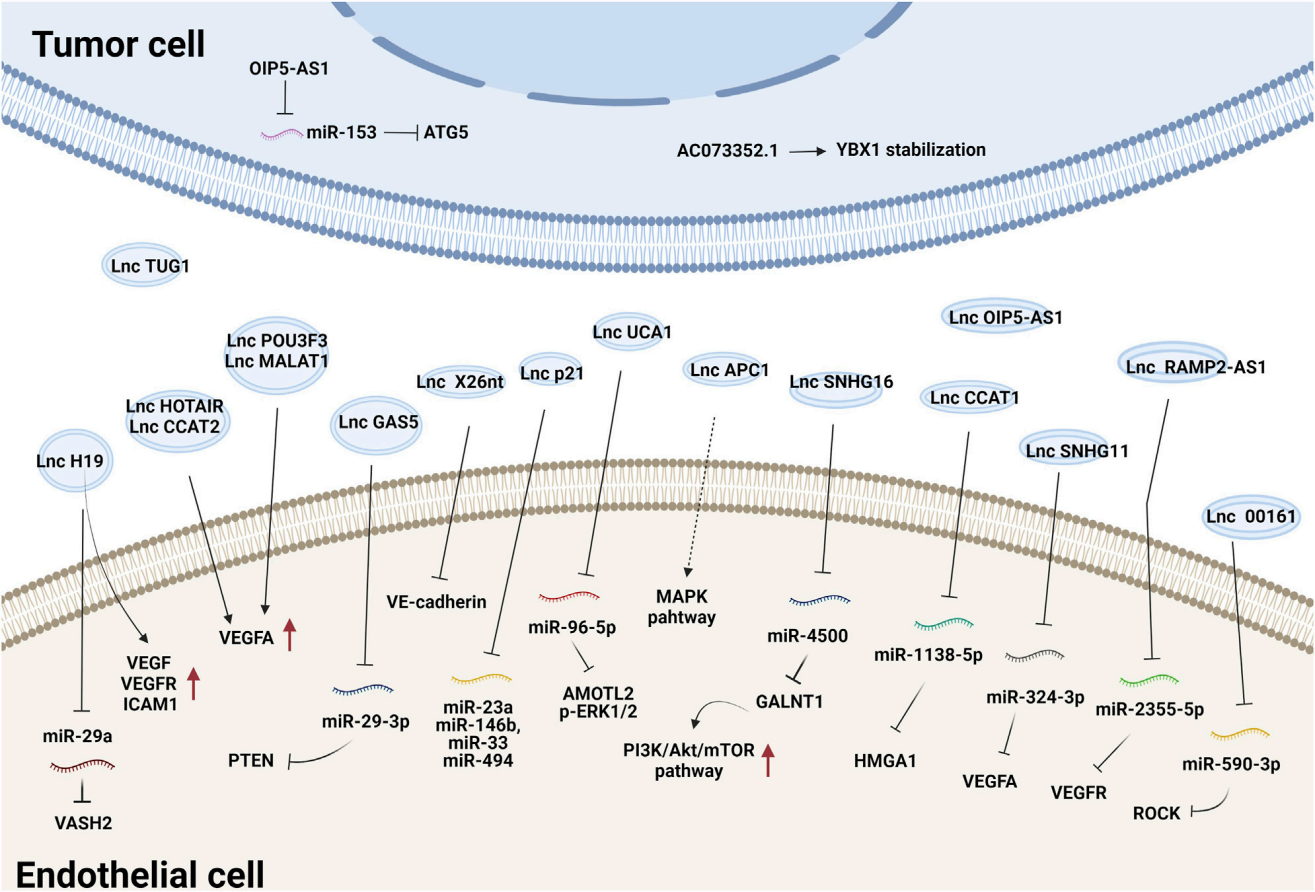
The effects and mechanisms of lncRNAs derived from tumor EVs on angiogenesis.

Tumor-secreted EV-derived lncRNAs can be transmitted to ECs where they promote the expression of proangiogenic genes and initiate angiogenesis by either binding to endogenous miRNAs or interacting with mRNAs and proteins (Ma et al., 2017; De Los Santos et al., 2019; Zhang et al., 2020). For example, lncRNA-H19 functions as an oncogene and is upregulated in multiple types of cancer (Iempridee, 2017). Exosomes derived from CD90^+^ liver cancer cells were found to be enriched in lncRNA H19 and promoted the angiogenic phenotype of human umbilical vein endothelial cells (HUVECs), probably by regulating VEGF and VEGFR1 expression (Conigliaro et al., 2015). Chondrosarcoma cell-derived exosomes containing lncRNA-RAMP2-AS1 promoted the proliferation, migration and tube formation of ECs by upregulating VEGFR2 by sponging miR-2355-5p (Cheng et al., 2020). LncRNA-UCA1 was highly expressed in exosomes derived from hypoxic pancreatic cancer (PC) cells and promoted angiogenesis and tumor growth by regulating the miR-96-5p/AMOTL2/ERK1/2 axis (Guo et al., 2020). PC-derived exosomal lncRNA SNHG11 promoted the expression of VEGFA by sponging miR-324-3p (Fang et al., 2021). Additionally, glioma-derived exosomal lncRNA-CCAT2 (Lang et al., 2017b) and lncRNA-POU3F3 (Lang et al., 2017a) enhanced angiogenesis by inducing VEGFA expression. LncRNA-APC1, a suppressor of angiogenesis, was significantly downregulated in colorectal cancer cell-derived EVs. It directly bound to and degraded Rab5b mRNA to decrease EV production and block the mitogen-activated protein kinase (MAPK) signaling pathway in HUVECs to suppress angiogenesis (Wang et al., 2019a). Together, these studies demonstrate that tumor exosomal lncRNAs regulate angiogenesis mainly by modulating VEGFA expression and the VEGF/VEGFR and MAPK pathways. The effects and mechanisms of other EV-derived lncRNAs on tumor angiogenesis are summarized in Figure 1 and Table 1.

#### 2.1.3 CircRNAs

**FIGURE 2:**
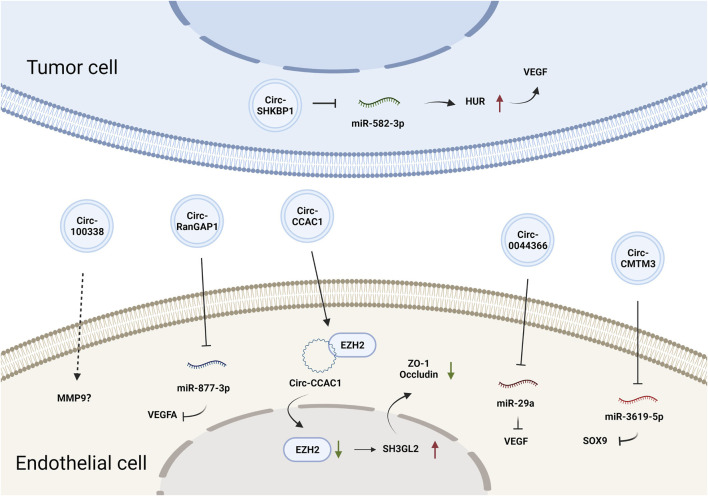
The effects and mechanisms of circRNAs derived from tumor EVs on angiogenesis.

CircRNAs constitute a class of endogenous ncRNAs that form a covalently closed loop without a 5′-cap or 3′-poly-A tail (Gan et al., 2021). They are produced by backsplicing protein-coding precursor mRNAs and regarded as variants of competitive endogenous (ceRNAs) that can sponge and thus inhibit the activity of miRNAs (Hansen et al., 2013). Accumulating evidence has demonstrated that circRNAs are involved in various biological processes by regulating gene expression at the transcriptional or posttranscriptional levels (Du et al., 2016). CircRNAs can also be loaded into EVs and mediate cell-cell communication. Circ-SHKBP1 in GC cell-derived exosomes promoted angiogenesis by sponging miR-582-3p and thus increased the expression of hu-antigen R (HUR), which regulated VEGF mRNA stability (Xie et al., 2020b). Circ-RanGAP1 in secreted exosomes derived from the plasma of GC patients and promoted GC progression by targeting the miR-877-3p/VEGFA axis (Lu et al., 2020). Additionally, circ-0044366/circ29, which is highly expressed in GC cell-derived exosomes, was delivered into ECs and sponged miR-29a to promote angiogenesis by upregulating VEGF (Li et al., 2021a). In summary, tumor EV-derived circRNAs affect tumor angiogenesis primarily by regulating VEGF expression. The effects and mechanisms of other EV-derived circRNAs on tumor angiogenesis are summarized in Figure 2 and Table 1.

### 2.2 EV-Derived Proteins and Tumor Angiogenesis

In recent years, researchers have identified thousands of proteins from different types of tumor-derived EVs, and some of these proteins were characterized with proangiogenic properties and can stimulate various steps in the angiogenic cascade. For example, EVs derived from colorectal cancer perivascular cells contained growth arrest specific 6 (Gas6) and promoted the recruitment of endothelial progenitor cells (EPCs) to tumors by activating the Axl pathway, thus leading to tumor revascularization after withdrawal of antiangiogenic drugs (Huang et al., 2021). VEGFA was carried in EVs derived from *ex vivo* cultured patient-derived glioblastoma stem-like cells and promoted angiogenesis of human brain ECs (Treps et al., 2017). Breast cancer cell-derived EVs contained VEGF_90K_, which was generated by VEGF_165_ crosslinking and triggered sustained activation of VEGFRs in ECs by interacting with heat shock protein 90 (HSP90) (Feng et al., 2017). Furthermore, EVs secreted by ovarian (ES2), colorectal (HCT116), and renal (786–0) cancer cells, in bodily fluids of tumor-bearing mice, and in ovarian cancer patient ascites could stimulate EC migration and tube formation. These responses were mediated by the 189 amino acid isoform of VEGF (VEGF_189_), which was bound to the surface of these EVs because of its high affinity for heparin (Ko et al., 2019). Collectively, these findings indicate that proangiogenic factors (e.g., Gas6 and VEGFA) and different subtypes of VEGF promote tumor angiogenesis through different mechanisms.

**FIGURE 3:**
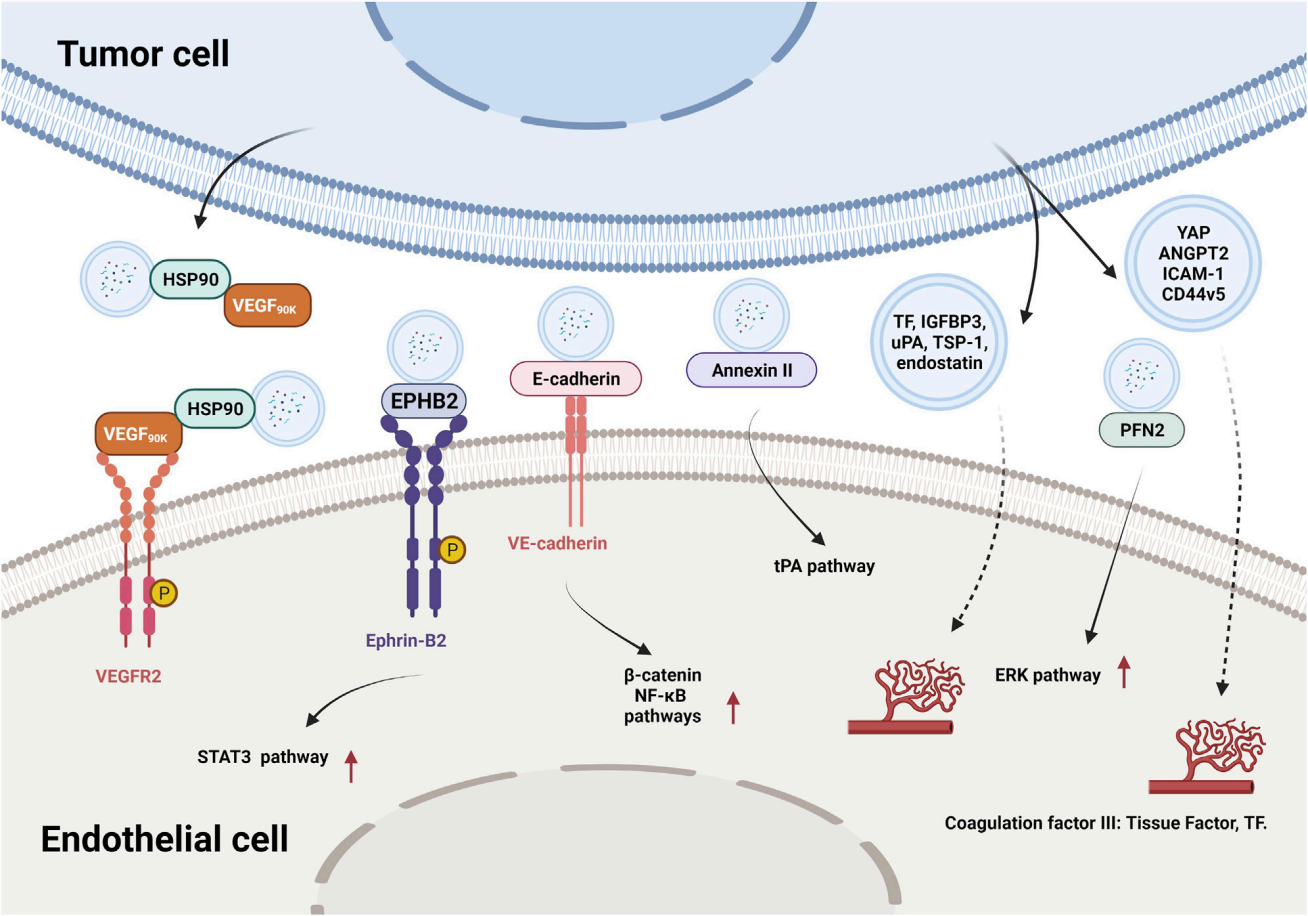
The effects and mechanisms of proteins derived from tumor EVs on angiogenesis.

In addition to conventional proangiogenic cytokines, other angiogenesis-related proteins have also been found in EVs. Ephrin type B receptor 2 (EPHB2) in small EVs derived from head and neck squamous cell carcinoma (HNSCC) activated ephrin-B reverse signaling and induced STAT3 phosphorylation in ECs, which promoted angiogenesis both *in vitro* and *in vivo* (Sato et al., 2019). Moreover, soluble E-cadherin, which was localized to the surface of exosomes derived from ovarian cancer (OV) cells, activated the *β*-catenin and nuclear factor-κB (NF-κB) signaling pathways by interacting with VE-cadherin on ECs, leading to angiogenesis *in vitro* and *in vivo* (Tang et al., 2018). Exosomal Annexin II secreted by breast cancer cells promoted tPA-dependent angiogenesis *in vitro* and *in vivo* (Maji et al., 2017). Wnt5A induced the secretion of exosomes containing proangiogenic proteins (e.g., VEGF and MMP2) and immunomodulatory factors (e.g., IL-8 and IL-6) by melanoma cells (Ekstrom et al., 2014). Additionally, other angiogenic proteins have been found in many cancer cell-secreted EVs, such as yes-associated protein (YAP) (Wang et al., 2019b), angiopoietin 2 (ANGPT2) (Xie et al., 2020a), profilin 2 (PFN2) (Cao et al., 2020), Dll4 (Sheldon et al., 2010), ANG, IL-6, IL-8, tissue inhibitor of metalloproteinases-1 (TIMP-1), TIMP-2, activating transcription factor 2 (ATF2), metastasis associated 1 (MTA1), and Rho associated coiled-coil containing protein kinase 1/2 (ROCK1/2) (Skog et al., 2008; Chan et al., 2015; Yi et al., 2015; Ikeda et al., 2021). More proteins in different types of tumor-derived EVs and their proangiogenic mechanisms are summarized in Figure 3 and Table 2.

**TABLE 2:** The effects and mechanisms of proteins derived from tumor EVs on angiogenesis.

Cargoes	Tumor types or donor cells	Recipient cells	Signaling pathways	Functions	References
Gas6	Perivascular cells from CRC	EPCs	Activation the Axl pathway	Revascularization	Huang et al. (2021)
VEGF_90K_	BC	HUVECs	VEGF_90K_-HSP90 complex	Proangiogenesis	Feng et al. (2017)
VEGF_189_	OC, CRC, ccRCC, OC patient ascites	HUVECs	Association with the surface of small EVs via heparin-binding	Proangiogenesis	Ko et al. (2019)
EPHB2	HNSCC	HUVECs	Ephrin-B2-STAT3 angiogenic signaling cascade	Proangiogenesis	Sato et al. (2019)
Soluble E-cadherin	OC	HUVECs	Activation of the β-catenin and NF-κB signaling pathways in ECs	Proangiogenesis	Tang et al. (2018)
Annexin II	BC	HUVECs	Activation of the tPA pathway	Proangiogenesis	Maji et al. (2017)
YAP	LC	HUVECs	—	Proangiogenesis	Wang et al. (2019b)
Coagulation factor III, IGFBP3, uPA, TSP-1, endostatin	HNSCC	HUVECs	Functional reprogramming and phenotypic modulation of ECs	Proangiogenesis	Ludwig et al. (2018)
ANGPT2	HCC	HUVECs	—	Proangiogenesis	Xie et al. (2020a)
PFN2	LC	HUVECs	Activation of the Erk pathway	Proangiogenesis	Cao et al. (2020)
ICAM-1, CD44v5	NPC	HUVECs	—	Proangiogenesis	Chan et al. (2015)

Abbreviations: urokinase type plasminogen activator, uPA; tissue plasminogen activator, tPA.

## 3 Extracellular Vesicles and Clinical Implications

As ncRNAs or proteins loaded in EVs can be distributed in various biofluids, such as blood, urine, tears, saliva, milk, and ascites (Keller et al., 2011), the ability to analyze their cargoes and levels in bodily fluids makes them promising biomarkers for cancer diagnosis and prognosis (Sun and Liu, 2014). Liquid biopsy is a noninvasive method of detecting precise information about the tumor environment/status, which can provide information prior to treatment (Rekker et al., 2014). Through liquid biopsy, numerous proangiogenic contents in EVs have been identified.

Similar to that on circulating free DNA or cell-free DNA and several oncoproteins, such as prostate-specific antigen (PSA) and alpha-fetoprotein (AFP), emerging evidence has suggested that EV-associated ncRNAs and proteins can serve as biomarkers and diagnostic, prognostic, and therapeutic targets in cancer patients.

The levels of serum miR-210 and serum-derived exosomal miR-210 were much higher in HCC patients than in healthy donors. A high level of miR-210 was associated with higher microvessel density in HCC patients (Lin et al., 2018). Increased expression of exosomal circRNA-100338 in the serum of HCC patients was associated with tumor growth and angiogenesis in primary and metastatic HCC. Exosomal circRNA-100338 can serve as a predictor of poor prognosis and lung metastasis in HCC patients following curative hepatectomy (Huang et al., 2020b). Serum exosomal Annexin II promoted angiogenesis, and a high level of serum exosomal Annexin II was associated with tumor grade, poor overall survival (OS), and poor disease-free survival in African-American women with triple-negative breast cancer (Chaudhary et al., 2020). Increased expression of lnc-UCA1 was positively correlated with microvessel density in PC tissues. Exosomal lnc-UCA1 levels were greatly increased in PC patient serum and were associated with tumor size, lymphatic invasion, late tumor node and metastasis stage, and poor OS (Guo et al., 2020). The elevated expression of metastasis associated lung adenocarcinoma transcript 1 (MALAT1) in exosomes derived from epithelial ovarian cancer (EOC) patient serum was significantly correlated with an advanced and metastatic phenotype and served as an independent predictive factor for the OS of EOC patients (Qiu et al., 2018). NSCLC patients with high levels of lncRNA-p21 in EVs derived from tumor-draining pulmonary veins exhibited shorter relapse-free survival and OS (Castellano et al., 2020). The level of circ-CCAC1 in the EVs in the serum of cholangiocarcinoma patients was significantly increased compared to that of patients with benign hepatobiliary disease, indicating that circ-CCAC1 in EVs may serve as a biomarker for cholangiocarcinoma (Xu et al., 2021). CRC patients with metastasis showed a higher level of miR-25-3p in exosomes than patients without metastasis (Zeng et al., 2018). The expression of miR-619-5p in exosomes was increased in the serum of NSCLC patients, indicating that miR-619-5p can serve as a diagnostic indicator (Kim et al., 2020). High levels of exosomal miR-1260b were associated with high-grade disease, metastasis, and poor survival in patients with NSCLC (Kim et al., 2021).

Moreover, prostate-specific membrane antigen (PSMA) has emerged as a specific prostate tumor biomarker in prostate tumor-derived exosomes. Ziaei et al. developed a novel biofunctionalized silica nanostructure to capture tumor-derived exosomes through the interaction of PSMA and its ligand TG97, providing a noninvasive approach for prostate cancer diagnosis (Ziaei et al., 2017). The company MiRXES performed a test to analyze the levels of 12 miRNA biomarkers linked to GC and calculated a cancer risk score for each patient (Kapoor et al., 2020). Another study indicated that the level of phosphatidylserine-expressing tumor-derived exosomes in the blood is a reliable biomarker for early-stage cancer diagnosis (Sharma et al., 2017).

## 4 Conclusion and Perspectives

Tumor angiogenesis plays a critical role in tumor growth and development, and antiangiogenic therapy has been frequently applied to the clinical treatment of multiple solid tumors. Among the generally known proangiogenic signaling pathways, miRNAs, lncRNAs, circRNAs, and proteins carried by tumor-secreted EVs have recently emerged as important modulators of tumor angiogenesis, acting through a variety of mechanisms, as described in this review.

Antiangiogenic therapy has been widely used for the treatment of various solid tumors and has conferred tremendous survival benefits to cancer patients (Teleanu et al., 2019; Lugano et al., 2020). Antiangiogenic drugs, such as bevacizumab, sorafenib, and regorafenib, inhibit tumor growth by suppressing angiogenesis primarily through blocking the VEGF/VEGFR pathway. However, many patients receive only modest survival benefits and develop acquired resistance to antiangiogenic drugs (Huijbers et al., 2016; Gacche and Assaraf, 2018). Drug resistance is one of the most important obstacles to treatment because it limits the clinical applications of antiangiogenic drugs, and the diseases still progress, which results in poor outcomes and unsatisfactory quality of life (Sennino and McDonald, 2012; van Beijnum et al., 2015). Since exosome-derived ncRNAs and proteins play important roles in tumor angiogenesis, targeting ncRNAs and proangiogenic proteins may be a potential therapeutic strategy to inhibit tumor angiogenesis.

Because a single miRNA, lncRNA, and circRNA species has the potential to regulate angiogenesis by modulating multiple targets, these ncRNAs hold great promise for use in therapeutic approaches to the treatment of tumor angiogenesis. However, in addition to tumors, ncRNAs significantly regulate the biological functions of normal cells, and systemic targeting of ncRNAs might affect physiological angiogenesis in normal tissues. Therefore, it is important to develop more specific therapeutic approaches based on angiogenesis-related ncRNAs. Moreover, EVs have turned out to be possible natural carriers of therapeutic agents with long half-time and non-immunogenic properties (Lakhal and Wood, 2011). These EV-based nanocarriers exhibit several advantages such as a high capacity for overcoming various biological barriers and high stability in the blood (Ha et al., 2016). However, the safety, specificity, and proficiency of this promising approach in clinical trials still remain more mysterious. EVs-based nanocarriers still face many challenges in clinical application.

In summary, this review provides deeper insight into the regulatory role of tumor-derived EVs on angiogenesis. Therefore, revealing the mechanisms of tumor-derived EVs on angiogenesis and seeking their potential as biomarkers and diagnostic, prognostic, and therapeutic targets in cancer patients will be popular research directions in the future.
